# Accuracy of online discussion forums on common childhood ailments

**DOI:** 10.5195/jmla.2018.355

**Published:** 2018-10-01

**Authors:** Alison Farrell

**Affiliations:** Public Services Librarian, Health Sciences Library, Memorial University of Newfoundland, St. John’s, NL, Canada

## Abstract

**Objectives:**

The research sought to determine if the health advice provided in online discussion forms aimed at parents of young children is accurate and in agreement with evidence found in evidence-based resources and to discover whether or not these forums are an avenue for misinformation.

**Methods:**

To determine which online forums to use, Google was searched using five common childhood ailments. Forums that appeared five or more times in the first five pages of the Google search for each question were considered. Of these forums, those that met the inclusion criteria were used. Data from a six-month time period was collected and categorized from the discussion forums to analyze the advice being provided about common childhood ailments. Evidence-based resources were used to analyze the accuracy of the advice provided.

**Results:**

Two discussion forums were chosen for analysis. Seventy-four questions from one and 131 questions from the other were health related. Data were not analyzed together. Of the health-related questions on the 2 forums, 65.5% and 51.8%, respectively, provided some type of advice. Of the advice provided, 54.1% and 47.2%, respectively, agreed with the evidence provided in evidence-based resources. A further 16.2% and 6.3% was refuted or was somewhat refuted by the evidence found in evidence-based resources.

**Conclusion:**

While roughly half of the health-related advice provided in online discussion forums aimed at parents of young children is accurate, only a small portion of the advice is incorrect; therefore, these sources are not a major concern for the spread of misinformation.

## INTRODUCTION

Many parents today go online to seek out advice and support when dealing with common childhood problems, diseases, conditions, ailments, and other issues. Sebelefsky et al. found that 94.4% of parents attending a pediatric outpatient clinic in Austria used the Internet to find health-related information [1]. This result agreed with a study by Moseley et al., who found that among parents at pediatric clinics in southeast Michigan, 96% used the Internet to find health information pertaining to their children [2]. In the general population, according to the Pew Research Center, 72% of American adults use the Internet to find health-related information [3]. However, as in the general population, there are varying levels of information literacy among parents, ranging from parents who trust everything they read online, with no critical appraisal of the information, to parents who seek only the highest-quality evidence and view the information with a high degree of skepticism. Therefore, it is important to understand the nature of the online health information that parents consider.

There are numerous avenues for obtaining health information online, with some being evidence-based and others being purely opinion based. One type of information source that often appears when searching the Internet for common childhood ailments—such as fever, leg pain, or pink eye—are online discussion forums. In Sebelefsky et al.’s study, of those parents who used the Internet for health-related information, 62% used health forums and communities [1]. These forums can range from being directed at a very particular audience, such as parents of children with type 1 diabetes, to broad forums on general parenting topics. In a systematic review conducted in 2014 examining how social media were used in health care, Hamm et al. found that almost all studies showed a high use of social networking sites, with discussion forums being one of the most widely used tools on the sites [4]. In a study of Hispanic mothers, discussion forums, specifically those on BabyCenter.com, were widely used because participants valued advice from others in similar situations or with similarly aged children [5]. One reason for trust in this type of site was that the answers were found to be similar to what one might see in books [5]. Similarly, in an examination of the discussion forum on the parenting website mumsnet, Doyle found that people used the forum to advise others to seek medical care, give interpretations of symptoms and possible diagnosis, provide advice to push for specialist care, and provide advice for self-care [6]. Doyle found that people tended to trust the information if several responders provided similar information.

One issue with discussion forums is that there is a high potential for the spread of misinformation due to the potential lack of accurate information [7–9]. Another issue leading to the spread of misinformation is the potential for a respondent to present patient-specific advice received from a health professional, which may not be appropriate for the original questioner [7]. Despite this potential for the spread of misinformation, Balkhi et al. found a high degree of trust placed in the community that populates the forums in a study of parents using an online discussion forum specific to type 1 diabetes [8]. By contrast, Bernhardt et al. found that participants were more reluctant to trust information found from other parents, specifically diagnosis and treatment advice [9].

The accuracy of advice provided in online discussion forums has rarely been assessed [7], though Henderson et al. claimed that forum websites were of a lower quality than medical-type websites or “not for profit” health websites. This analysis was based on characteristics of the sites such as reading level, site design, and transparency of information source [10], rather than the content of the advice being provided. By contrast, Cole et al. examined 3 different forums and assessed the quality of information presented on 3 different topics: HIV, diabetes, and chickenpox. They found that most information was of reasonably good quality and concluded that discussion forums can be valuable places to find health-related information [11]. In a specific forum for content related to type 2 diabetes for retired people, most advice (74%) was in complete agreement with best practice guidelines, while only 9% was inaccurate or potentially misleading [12].

If looking only at the studies by Cole et al. [11] and Hoffman-Goetz et al. [12], it could be assumed that online discussion forums are sources of reliable information; however, these studies were both small and limited to very specific conditions. Because there are very few studies examining the accuracy of the information presented and the studies that do exist focus on specific conditions, no general conclusions can be drawn at present about the quality of advice provided on all-purpose forums.

The focus of this study was to examine general online discussion forums with a health section aimed at parents of young children to determine if the advice provided agreed with information found in evidence-based, point-of-care resources. The results could be used to inform health sciences librarians while they help consumers find appropriate, accurate, and reliable information.

## METHODS

As per article 2.2 of the Tri-Council Policy Statement: Ethical Conduct for Research Involving Humans (TCPS2 (2014)) [13], this research was exempt from ethics board approval.

### Forum selection

Inclusion criteria were developed to determine which online discussion forums to include in data analysis. Because the intention was to examine publicly available sites that any parent could access, only public forums that did not require a login to read posts were considered. Additional inclusion criteria were a target population of parents, posts written by the general public rather than health professionals, and the presence of a forum section specifically aimed at children’s health.

To select the forums for analysis, five typical children’s health topics were selected and searched using Google, which was chosen because it is the most commonly used search engine [14]. The topics were: a three-year-old with fever and sore legs, an eighteen-month-old teething, a four-year-old with pink eye, a two-year-old with croup, and a six-month-old with a rash on their belly. These topics were chosen using personal experience as well as informal discussions with colleagues and friends who were parents. The investigator’s browsing history and cache were cleared to eliminate any searching bias. While searchers tend to only look at the first one to three pages of search results [15], all forums found in the first five pages of the Google results for each of the five topics were recorded to ensure comprehensiveness in results. This resulted in eighteen forums, which were considered against the inclusion criteria. A total of two forums (BabyCenter.com and WhattoExpect.com) met all of the criteria and were, thus, used in data analysis.

### Data collection

All questions in a six-month time period (June 2016–November 2016) that had at least one answer were extracted and used for data analysis. Due to the structure of the forums, no software could be found to automatically extract questions and answers; therefore, questions and answers were extracted from the forums using manual copy and paste into Microsoft Excel for analysis. The structure of the posts differed between the forums, resulting in slightly different data collection methods. Due to this difference, data from different forums could not be pooled and were analyzed separately.

#### BabyCenter.com

The BabyCenter.com website has several sections varying from expert advice to community posts. As the focus of this study was to evaluate the public discussion posts written by parents rather than health professionals, the “Mom Answers” section was examined. This section was further broken down into subsections. Although the original intention was to use only the “Children’s Health” subsection, upon examination, this subsection contained very little data, and numerous health-related questions were also found in other subsections of the website. Therefore, the subsections used were: “Baby,” “Toddler,” “Preschooler,” “Big Kid,” and “Children’s Health.”

#### WhattoExpect.com

The WhattoExpect.com website has several sections varying from professional advice, to videos, to community posts. For this website, the community section was examined, with the “Baby’s 1st Year,” “The Toddler Years,” “Preschooler Years,” “School-Age Years,” and “Kids Health” subsections used for data extraction.

### Data analysis

Content analysis was used to code all questions into general topics. Any questions that were coded as health were further coded into specific health topics (e.g., croup, congestion, rash). Answers were also coded using content analysis into type of answer (e.g., support, advice, follow-up questions). Any answers that provided advice were then searched in evidence-based, point-of-care resources to determine if the advice was supported or refuted by the evidence provided. Evidence-based resources were chosen based on availability of access from the author’s institutional subscriptions and included DynaMed, UpToDate, RxTx, and Natural Medicines. Advice response categories and their explanations are shown in Table 1.

**Table 1:** Advice categories with descriptions

Advice category	Abbreviation	Explanation
Supported	S	Advice was fully supported by evidence
Somewhat supported	SS	Advice was partially supported by evidence
Refuted	R	Advice was fully refuted by evidence
Somewhat refuted	SR	Advice was partially refuted by evidence
No evidence found	NEF	Advice could not be supported or refuted by evidence because no evidence was found
Not applicable	—	Advice focused on prevention rather than treatment

The “Not applicable” category included topics surrounding vaccinations, sleep, nutrition, pregnancy advice, and non-health-related advice. As the focus of this study was on the diagnosis, treatment, and management of conditions, advice about preventative measures was excluded from analysis. Although an additional category of advice might have been possible (i.e., advice was partly supported by evidence and partly refuted by evidence), there were no occurrences of this type of advice.

## RESULTS

A total of 492 questions and 777 responses from BabyCenter.com were examined (supplemental Appendix A). Of these, 74 questions were health related and were accompanied by a total of 113 responses. Other types of questions pertained to topics such as sleep, behavior, and nutrition. Health topics ranged from very common ailments, such as constipation and congestion, to more rare ailments, such as visible veins and hernia. Of the 113 responses, 74 (65.5%) provided advice, 30 (26.5%) suggested that the original questioner seek medical attention, 26 (23.0%) offered a diagnosis of some sort, and 30 (26.5%) offered support, asked follow-up questions, or provided comments such as sympathizing with the original questioner. Percentages do not add up to 100 since some responses contained more than 1 type of response (e.g., a responder may have offered a diagnosis and suggested that the questioner seek medical attention).

A total of 587 questions and 3,445 responses from WhattoExpect.com were examined (supplemental Appendix B). Of these, 131 questions were health related and were accompanied by a total of 650 responses. Of the 650 responses, 337 (51.8%) offered advice, 89 (13.7%) suggested that the questioner seek medical attention, 57 (8.8%) offered a diagnosis, and 278 (42.8%) offered support, asked follow-up questions, or provided comments.

Table 2 shows the numbers of all questions and answers in various categories from BabyCenter.com and WhattoExpect.com, and Table 3 shows the numbers of health-related topics from both sites. Categories or topics were placed in “other” if there were fewer than five questions in total from both sites in that particular category or topic. Some questions were coded as containing more than one health topic (e.g., a question about both fever and congestion).

**Table 2:** Categories of questions and responses on BabyCenter.com and WhattoExpect.com

Question category	BabyCenter.com		WhattoExpect.com	

	Questions	Responses	Questions	Responses
Health	74	113	131	650
Sleep	67	114	105	580
Behavior	49	72	36	191
Development	32	47	38	193
Nutrition	102	149	96	559
Potty training	17	20	15	80
Adoption	23	53	0	0
Baby equipment	15	23	63	370
Child care	13	22	17	147
Pregnancy worries	11	19	0	0
Support	0	0	20	116
Other	64	120	71	557

**Table 3:** Health topics of questions on BabyCenter.com and WhattoExpect.com

Health topic	BabyCenter.com		WhattoExpect.com	

	Questions	Responses	Questions	Responses
Bowel movements (e.g., constipation, diarrhea, abnormal color)	16	29	15	74
Reflux/gas/vomiting	8	14	10	68
Common cold/congestion	3	4	12	63
Rash/eczema	7	7	11	53
Ear infection/ear pain	1	2	4	16
Fever	3	4	3	16
Preventative hygiene	1	1	6	36
Vaccinations (considered not applicable)	2	2	6	38
Other	34	40	64	286

**Figure 1:**
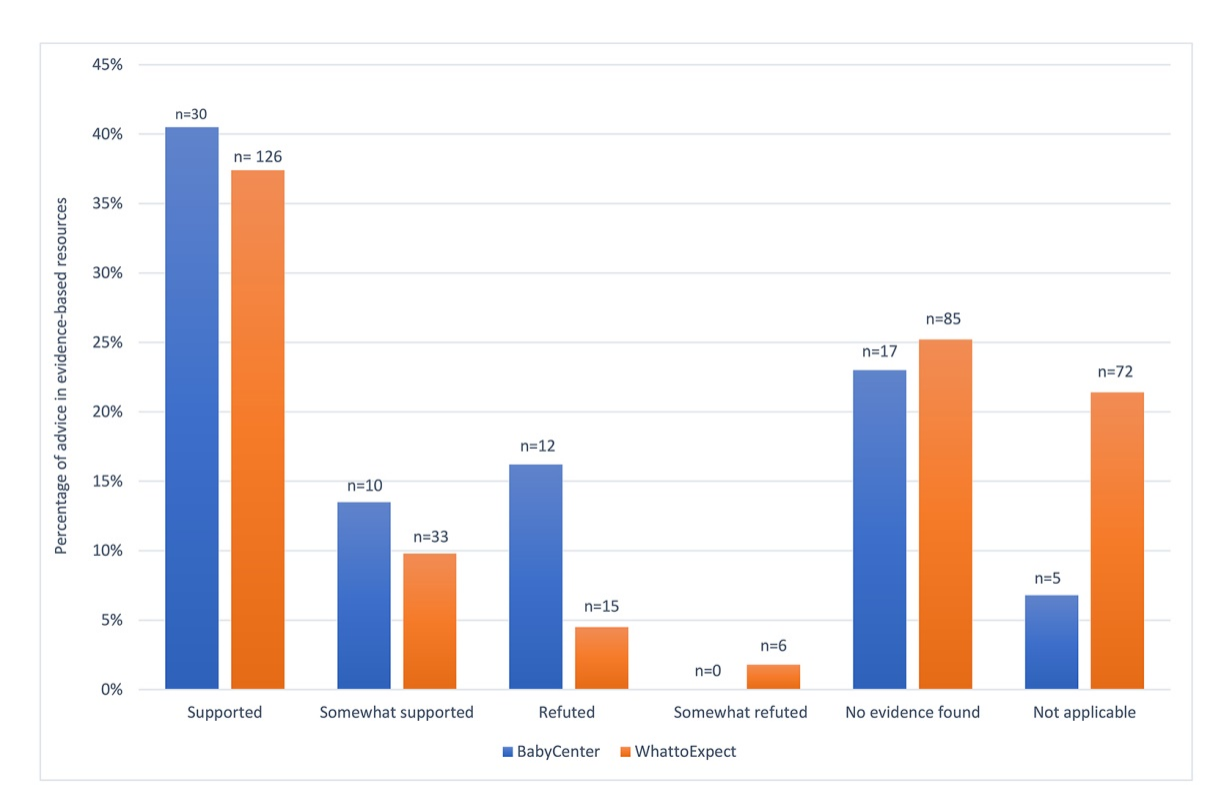
Level of agreement of discussion forum advice to medical evidence found in evidence-based resources

Figure 1 shows how the overall advice on each website compared to evidence found in DynaMed, UpToDate, RxTx, and Natural Medicines, and Table 4 shows the breakdown of specific health topics and how they compared to the medical evidence. Topics were included in Table 4 if there were five or more questions on the specific topic in both databases combined.

**Table 4:** Breakdown of specific health conditions according to the level of agreement with medical evidence found in evidence-based resources

Health topic	BabyCenter.com					WhattoExpect.com				

	S	SS	R	SR	NEF	S	SS	R	SR	NEF
Bowel movements	12	2	6	1	4	22	4	3	0	7
Reflux/gas/vomit	7	1	0	0	1	0	2	0	0	36
Cold/congestion	3	1	0	0	0	28	2	2	0	2
Rash/eczema	3	2	0	0	2	14	3	0	0	16
Fever	2	0	0	0	0	5	2	0	0	1

S=fully supported, SS=somewhat supported, R=refuted, SR=somewhat refuted, NEF=no evidence found to support or refute.

**Figure 2:**
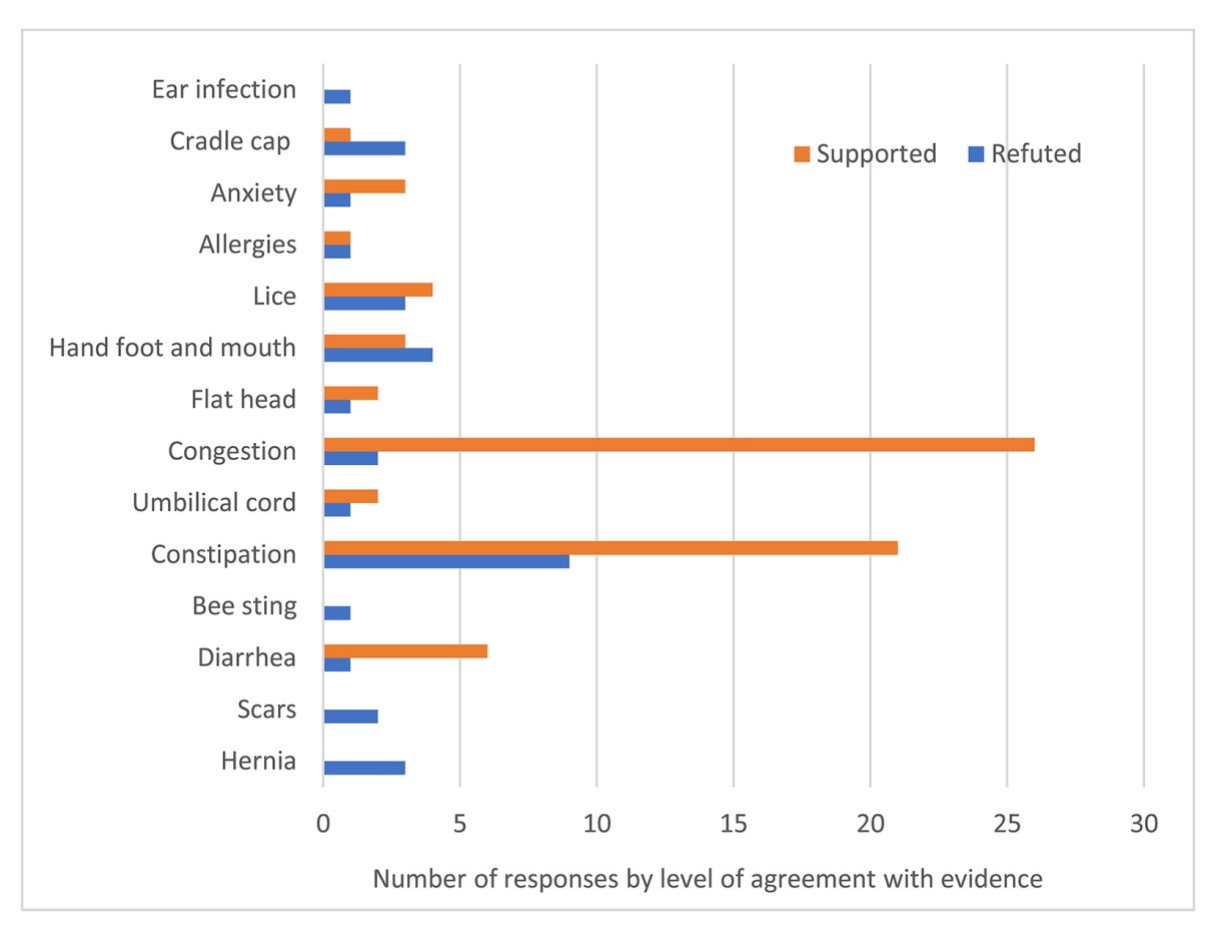
Topics with advice that was refuted by medical evidence from evidence-based resources

Only 16.2% and 6.3% of advice on BabyCenter.com and WhattoExpect.com, respectively, was refuted or somewhat refuted by the medical evidence found in evidence-based resources. The topics on which people provided advice that was refuted for both forums combined are shown in Figure 2. Interestingly, for most topics that had advice that was refuted by medical evidence, there were other responses on the same topic that provided advice that was supported by medical evidence, indicating that these topics are not misunderstood by all responders.

## DISCUSSION

This study sought to determine if health advice that is provided in online discussion forums aimed at parents of young children agreed with information found in evidence-based, point-of-care resources. Using the forums BabyCenter.com and WhattoExpect.com, it can be seen that there is a wide variety of health topics that parents are discussing online. The topics are so varied that there are little data for any particular topic, and therefore, the present results cannot provide definitive conclusions. However, it is still possible to draw some basic inferences.

The most commonly discussed topic was bowel movements, for which the advice provided was well in agreement with medical evidence. Of 61 responses that provided advice about bowel movements (i.e., constipation, diarrhea, and discoloration), more than half (66%) agreed with the medical evidence, while only 16% disagreed. Other commonly discussed topics included the common cold or congestion and eczema or rash. A majority of responses providing advice about the common cold or congestion agreed with medical evidence (90%), while only 5% disagreed. However, the advice provided about rash or eczema did not have the same level of agreement, with just over half (55%) of the advice agreeing with medical evidence. However, there were no responses that were refuted by medical evidence. Much of the advice surrounding rashes involved natural medicines, with no evidence found to support or refute the advice.

One of the aims of this research was to identify areas of educational need. It was hoped that health topics that were not well understood would be identified to determine where educational efforts should be aimed. From this small study, it can be concluded that while users of online discussion forums aimed at parents of small children do not always provide advice that is supported by medical evidence, they are not providing a great deal of incorrect advice but rather are providing advice that cannot be supported or refuted using evidence found in medical evidence-based resources. While less than half of the responses providing advice agreed with medical evidence, only a small proportion disagreed. This might indicate that although people do not necessarily know what to do to treat or alleviate a particular ailment, they generally are not providing harmful advice to others. This aligns with findings from both Cole [11] and Hoffman-Geotz [12] that most information in online discussion forums appears to be fairly accurate and not a cause for concern.

One potential for the spread of misinformation would likely be found in the responses that offer a diagnosis; however, there was not a large proportion of these responses, with only 23% of responses on BabyCenter.com and 9% of responses on WhattoExpect.com offering a diagnosis. In a future study, it would be interesting to examine the diagnoses being offered to determine if they could, in fact, be harmful.

There were two notable differences between the forums. Respondents on BabyCenter.com were more likely to either offer a diagnosis or to tell the original questioner to seek medical attention than responders on WhattoExpect.com, although it is not clear why this is the case.

While a significant portion of the advice provided on BabyCenter.com and WhattoExpect.com (54% and 47%, respectively) either agreed or somewhat agreed with medical evidence, a great deal of the advice could not be supported or refuted using DynaMed, UpToDate, RxTx, or Natural Medicines (23% and 25%, respectively). This could be due to the fact that only evidence-based resources were used to find the evidence. As no single studies were examined, if the evidence had not yet made it into 1 of the 4 resources used in this study, the response would have been deemed to have no evidence found. Another reason for this might be that a good deal of the advice surrounded the use of natural remedies, which did not have much medical evidence. While Natural Medicines was used, much of the advice was in the category of “insufficient reliable evidence to rate,” meaning that while a treatment might work, not enough research has been conducted to prove its efficacy.

### Limitations

This small study was designed to determine if there are obvious areas of educational need based on findings from online discussion forums designed for parents of young children. Due to the nature of the study, it has some inherent limitations. First, the data analysis was performed by one researcher; therefore, there was no inter-rater reliability in the coding of responses. However, a small set was first analyzed to determine if the coding system would work. The author then recoded the same set to check that the coding was consistent. Second, when determining if the advice was supported or refuted by medical evidence, it might have been useful to have a physician or other health professional help to make the judgments. As agreement with medical evidence was instead judged by a librarian to the best of their knowledge and ability, some misinterpretation may have been possible. Lastly, WhattoExpect.com was missing two months of posts for one of the five sections, with no explanation for this absence.

### Future research

During the course of this study, a number of issues came up that were not in the scope of the current research. This study looked only at posted questions that had responses. It would also be interesting to examine questions with no responses to determine if there are any unaddressed areas, which could indicate that users of the forums do not have any knowledge of those topics. While not examined in this study, it was interesting to note that few respondents provided sources of information for the advice they provided. It may be useful to consider what sources of information these respondents are providing, if any at all.

There were a number of responses for which no evidence was found to support or refute the claim. Though not recorded, it seemed that a great deal of the advice focused on natural treatments or products, which are generally lacking in medical evidence. It would be interesting to look at these responses again once more evidence is available. Lastly, it would also be of interest to compare the advice with information found on reputable consumer health websites such as WebMD or MedlinePlus. In this study, advice was only compared to resources that are classified as evidence-based on how they gather and display information, for example, providing levels of evidence and links to references.

## CONCLUSION

Six months of data from two different online discussion forums, BabyCenter.com and WhattoExpect.com, were mined and analyzed to determine if users of those forums have a good understanding of how to treat common childhood ailments. While there were not enough data to draw any definite conclusions, the results point to a general understanding of the common cold or congestion, constipation or other bowel movement concerns, and eczema or rash. There were no areas that stood out as generally misunderstood, suggesting that these types of forums do not contribute to the spread of misinformation. However, much of the advice provided on these forums could not be verified through the evidence-based resources DynaMed, UpToDate, RxTx, or Natural Medicines.

## SUPPLEMENTAL FILES

Appendix A BabyCenter.com data

Appendix B WhattoExpect.com data
